# Coordination and modularization: the experience of the joint prevention and control mechanism to COVID emergencies in China

**DOI:** 10.3389/fpubh.2024.1244769

**Published:** 2024-04-11

**Authors:** Jingjing Yan

**Affiliations:** College of Philosophy, Law and Political Science, Shanghai Normal University, Shanghai, China

**Keywords:** COVID, joint epidemic prevention and control mechanism, social network analysis, public health emergency, China

## Abstract

**Background:**

The integration of disparate emergency resources and the improvement of emergency response teamwork are the underlying trends and shared requirements for building resilience in an era of multiple global public health crises.

**Objective:**

This study investigated the emergency response with emergency collaboration networks of each functional module and the overall Joint Epidemic Prevention and Control Mechanism (JPCM) network in China’s COVID outbreak prevention and control.

**Methods:**

The study employed a scholarly framework of *“the integration of JPCM coordination and emergency collaborative modularization”* to explore the attributes of JPCM using social network analysis. The data were obtained from administrative records from JPCM’s official website, spanning January 2020 to December 2022.

**Results:**

The study examined the JPCM coordination and found several functional working modules of JPCM, such as Interrupt Spread, Manage Supply, Medical Rescue, Restore Work and Production, and Implement Responsibility modules. The network structure indicators showed that the Manage Supply module had the most extensive network connectivity, the shortest communication distance, and the most consistent collaboration. The E-I index of the overall JPCM network and the Manage Supply network were − 0.192 and − 0.452, respectively (at *p* < 0.001 and *p* < 0.05), indicating more internal relationships than external relationships. The E-I index of the Medical Rescue and Implement Responsibility collaboration networks were 0.122 and 0.147, respectively (at *p* < 0.001 and *p* < 0.05), indicating more external relationships than internal relationships. The QAP regression analysis showed that the most vital driver on the overall JPCM network was the Interrupt Spread module, followed by the Implement Responsibility and Medical Rescue modules.

**Discussion:**

The Interrupt Spread module initiated emergency coordination with most departments and agencies. The Manage Supply module ensured the flow of medical supplies and survival essentials, while the Medical Rescue module addressed the core aspects of the health emergency response. The Restore Work and Production module repaired the halt in production and livelihoods caused by the outbreak, strengthening and developing emergency coordination and roles across emergency organizations. The Implement Responsibility module provided more heterogeneous emergency response resources for the overall JPCM coordination, complementing the COVID cross-organizational emergency response coordination.

**Conclusion:**

The study on the JPCM case in China improves public health emergency management and aids informed decision-making.

## 1 Introduction

Efforts to increase knowledge of the frameworks for COVID emergency management have been ongoing in many countries (1–6). For example, public health departments in developed countries can comprehend comprehensive plans and response frameworks by combining response planning efforts during the outbreak, such as surveillance, epidemiology, laboratory, community mitigation, medical care, vaccine, risk communication, and so on (7, 8). Even though they have robust infrastructure, formal procedures, and resources to deal with the emergency, some countries still struggle to solve the issue (1). The study suggests this is due to many existing flaws extending beyond traditional health emergency management systems (2). From this perspective, structural issues in emergency management have become apparent according to studies on COVID response experience (3).

Notably, due to the structural problems in government emergency management systems that have been led by the disruptive events of the COVID emergency, new challenges with ripple effects were mounting rapidly as well (9). Especially when the viral epidemics appear to be in transition, many governments face challenges in prioritizing emergency response plans and optimizing measures for future surges (4). In investigating China’s COVID emergency response measures, apart from the typical systems that have emerged and accumulated due to socio-economic and political development, most have been explicitly developed based on lessons learned from emergency relief efforts in recent decades (10). The government keeps changing how emergency management agencies and operations work so that material, organizational and system resources can be combined and utilized as quickly as possible.

According to scholars following China’s most recent policy developments, the Joint Epidemic Prevention and Control Mechanism (JPCM) has become an essential component of COVID emergency management in this country (11). The official definition of the JPCM is a multi-ministerial coordination working mechanism established by the State Council of China. The Chinese government has responded to the increasing scale of comprehensive emergencies by enhancing JPCM. This has entailed the establishment of cross-organizational collaborative emergency response networks, cross-level governmental interaction and coordination channels dedicated to COVID response, and the developing of extensive grassroots community mobilization capacities. JPCM operates at various levels of government hierarchy across the country, which are supported by the General Office (GO) (also called emergency rescue headquarters) as a standing department (5, 6). In this manner, cross-level collaboration entities have evolved into a loosen-structured mechanism that divides tasks and assigns them to JPCMs at different levels. Then, each JPCM at a given level facilitates emergency health response tasks by promoting cross-organizational collaborations. As a critical case in emergency management, JPCM provides a complement for studying the advancing emergency management systems.

On this basis, it is essential to discuss JPCM’s response to COVID emergencies to complement a more comprehensive emergency management system. This study aimed to understand public health emergency management from the perspective of cross-organizational collaborative relationships and collaboration patterns in JPCM emergency response. This paper began with a literature review on collaborative emergency management. The study then analyzed the network structure of the JPCM emergency response network and the modular collaborative network using social network analysis. It also examined the cross-regional characteristics of the network using the E-I index. Finally, the impact of different emergency collaboration modules on the overall JPCM network was analyzed through QAP correlation analysis. Specifically, the text focused on three academic questions: (1) What network parameters defined the JPCM emergency response coordination? (2) Which modules participated in integrating JPCM emergency response coordination, and what kind of linkages were established among entities from various jurisdictions during the integration process? (3) How did the functional modules of emergency response impact the development of JPCM emergency response coordination? By answering these questions, this manuscript showed that in JPCM emergency response coordination, the Interrupt Spread module initiated emergency coordination with most departments and agencies. The Manage Supply module ensured the flow of medical supplies and survival essentials, while the Medical Rescue module addressed the core aspects of the health emergency response. The Restore Work and Production module, by repairing the halt in production and livelihoods caused by the outbreak, allowed coordination and roles between emergency organizations to be strengthened and developed. The Implement Responsibility module provided more heterogeneous emergency response resources for the overall JPCM coordination, complementing the COVID cross-organizational emergency response coordination.

## 2 Literature review

Scholars have researched cross-organizational emergency collaboration from various perspectives.

(i) *Collaboration in emergency management.* Generally speaking, several academics have noted that the field and profession of emergency management have evolved into one that is more collaborative (12–15). This entailed addressing issues that were large in scope, affected a significant population, or needed enormous resources beyond the traditional hierarchical boundaries (16–18). According to Waugh and Streib, the conventional bureaucratic, hierarchical paradigm has changed into a more flexible and dynamic network model that facilitates cooperation across several organizations, the government, and other sectors (19, 20). Different roles or players must carry out the decision’s subtasks in order for coordination to occur. Both horizontal and vertical cooperation were necessary for an efficient response to major catastrophes, enabling various forms of cooperation between the federal, state, and local governments (21). This brought us to the work of Bullock et al., who described inter-governmental relations as how various tiers of government coordinated and collaborated, focusing on cross-organizational collaboration for the effective implementation of policy decisions (22).
(ii) *Networks in emergency management.* Researchers studying disasters have shown interest in the network approach while examining emergency response activities (23–25). According to studies, creating and maintaining emergency management networks was crucial to modern disaster management techniques. One effective tool for reducing natural hazards and disasters was policy—networks and partnerships (13, 21). Researchers have employed a social network analysis approach to determine critical players, examine inter-organizational relationships, evaluate network architectures, compare official emergency management plans with pre-existing networks, and evaluate the effectiveness of emergency management networks (24–27). According to the study, the degree of accuracy in the collective judgments of network participants was considerably and positively associated with the centrality of the leading agency. Agencies possessing important positions in communication networks also wield more significant effects (28). The features of networks, which showed how much each member retained their independence and autonomy, dictated where those networks fall on a continuum (29). Additionally, as a disaster response mechanism, Kapucu et al. used networks, partnerships, and collaborations in inter-governmental connections (30).
(iii) *Entities in emergency response.* Organizations were so dependent on one another when it came to cooperation that, as Mandell noted, the collaboration process might result in creating a new entity (29). Kapucu et al. noted in their study that certain areas used the Emergency Operations Center as the central hub for response operations. It was a place where representatives from multiple sectors came together to ensure the timely distribution of critical information, including public safety, fire and rescue, law enforcement, city, local health agencies, and local water districts (30). Press conferences were held to inform the local population of the current situation and provide additional instructions. In order to support the community, public, private, and non-profit organizations align themselves with each other according to their goals and communicate through the Emergency Operations Center to work together in a coordinated way (30).

JPCM for COVID emergency response in China, as a critical case in emergency management, provided a complement for studying the method of collaboration between emergency organizations. JPCM remained prominent in the Chinese government’s emergency management apparatus facing the COVID recovery phase (11). Hence, there was a need for a differentiated investigation of how emergency management coordinating organizations among JPCM enable speedy reaction during sizeable public health emergencies in a country.

## 3 Materials and methods

### 3.1 Research design

This study gave a research framework for *“the integration of JPCM coordination and emergency collaborative modularization”* to evaluate the experience of cross-organizational networks to COVID emergencies and address theoretical research concerns. The following were the primary components of the research framework:

1. The emergency organizations participating in COVID cross-organizational emergency networks were organized by collecting and sorting data according to international, national, provincial and municipal, cross-regional, residential, third sector, and cyber jurisdiction criteria. Among these groups of emergency organizations, the World Health Organization, which monitored risks to global public health and coordinated responses to a wide range of health emergencies, was an example of an international emergency organization. National emergency organizations were a collection of central government agencies and departments. Some examples of national emergency departments included the National Health Commission, the Ministry of Emergency Management, and the Ministry of Public Security. Provincial and municipal emergency organizations were the collection of provincial and municipal agencies and departments that had jurisdiction over the location where the emergency occurred. Some examples of provincial and municipal emergency organizations included the Provincial JPCM, the Municipal JPCM, the Provincial Health Commission, and the Municipal Health Commission. Cross-regional emergency organizations, such as Disaster Medical Assistance Teams (DMATs), provide rapid-response medical rescue when public health and medical emergencies overwhelm the resources available in a region. Residential emergency organizations included the area’s streets (townships), communities (villages), and hospitals. Third-sector emergency groups pursued specific goals, frequently associated with particular social and political perspectives. In this study, one such association was the China Welfare Institute, which served as an example of one that can participate in the production and livelihood aid of people whose lives have been disrupted due to major natural disasters or severe public accidents. Cyber emergency organizations referred to the Network Resiliency Platform, where ICT regulators, policymakers, and other interested parties can share information. They viewed what initiatives and measures have been implemented to ensure that communities and individuals remained connected during the COVID-19 crisis.
2. The study examined the structure and characteristics of the overall JPCM coordination and the modular emergency collaboration network. The cross-organizational collaborative relations between emergency organizations were extracted, and the corresponding matrix was created. As a result, the overall JPCM coordination, Interrupt Spread, Manage Supply, Medical Rescue, Restore Work and Production, and Implement Responsibility collaboration networks can be derived. Moreover, these networks’ network density, centralization, average path length, network cohesion, and clustering coefficient were evaluated. It was then possible to derive the characteristics of network intensiveness, aggregation trend, network connectivity, and network independence. In addition, the overall JPCM and the functional modules were compared in terms of the similarities and differences that exist.
3. The external–internal (E–I) index was presented to discuss the flow of resources and the interaction between emergency organizations operating in different jurisdictions. Additionally, in the overall JPCM and module emergency collaboration networks, the collaborative trends of the international, national, provincial and municipal, cross-regional, residential, third sector, and cyber emergency organizations were compared.
4. The quadratic assignment process (QAP) analysis was used to conduct correlation and regression analyses between the emergency modules and the overall JPCM coordination matrix. The study examined how the emergency modules contributed to the development of cross-organizational emergency collaboration and explained how the emergency collaboration within each module affected the overall JPCM coordination.

### 3.2 Methods of network analysis

The study considered emergency organizations participating in COVID cross-organizational emergency collaboration as network actors. It used the links between actors to represent the collaborative relationships between emergency organizations. After that, the networks for COVID emergency collaboration can be mapped. To obtain a comprehensive enumeration of the organizations participating in COVID emergency response and their corresponding abbreviations, please refer to Supplementary Appendix.

Network density ( D ) is the ratio of the actual number of node connections to the theoretical maximum number of connections (23, 31). Equation (1) can calculate D values for a network with n nodes and m lines connecting them.

(1) $$D=\frac{2m}{n\left(n-1\right)}$$

where n and m are the numbers of nodes and connections between nodes in the network, respectively.

Network diameter ( ND ) and average path length ( APL ) measure the communication distance between emergency organizations in the emergency response coordination network and indicate the network’s overall connection. The network diameter measures the distance between the most distant emergency response groups that are collaborating. The average path length equals the average collaboration distance between all emergency organizations (23). The expressions for ND and APL can be found in the following Equations (2) and (3).

(2) $$ND=L_{\text{max}}$$

(3) $$APL=\frac{\sum_{i=1}^{n}L_{i}}{n}$$

where $L_{\text{max}}$ denotes the maximum distance between nodes; $L_{i}$ represents the distance between each pair of nodes; and n is the number of nodes in the network.

Network centralization ( C ) indicates the degree to which a network tends to cluster around its central node, whose values are computed using Equation (4). There are three types of network centralization: degree centralization, betweenness centralization, and closeness centralization (23, 32). This study employs degree centralization to determine whether the emergency response coordination network has a central emergency organization.

(4) $$C=\frac{\sum_{i=1}^{n}\left(C_{\text{max}}-C_{i}\right)}{\max\left[\sum_{i=1}^{n}\left(C_{\text{max}}-C_{i}\right)\right]}$$

where $C_{\text{max}}$ for the highest possible centrality value at each network node; $C_{i}$ for the centrality of node i .

The clustering coefficient ( CC ) is a metric to assess how well organizations in an emergency response coordination network are developing stable collaborative or modular organizational relationships (32). Equations (5) and (6) presents the density coefficient of node $n_{1}$ ( $DC_{n_{1}}$ ) and the CC of the network.

(5) $$DC_{n_{1}}=\frac{2u}{k\left(k-1\right)}$$

(6) $$CC=\frac{\sum_{i=1}^{n}DC_{n_{1}}}{n}$$

where n is the number of network nodes, $DC_{n_{1}}$ is node ${n_{1}}^{’}$ s density coefficient, k is the number of nodes immediately surrounding node $n_{1}$ , and u is the total number of connections between nodes immediately surrounding node $n_{1}$ .

#### 3.2.1 External–internal (E–I) index

The study introduces the E-I index to explain the linkages of interaction and resource flow among the international, national, provincial and municipal, cross-regional, residential, third sector, and cyber groups. The E–I index measures the relationship between factions and the number of factions in a network (33). The relationships between groups (external links, or ELs) and within each group (internal links, ILs) make up the cross-organizational emergency collaboration network’s primary relationships. Specifically, the E–I index is computed using Equation (7). The E–I index has a value range of [−1, +1] (34).

(7) $$E-I=\frac{ELs-ILs}{ELs+ILs}$$

where EL and IL stand for the number of external and internal links, respectively.

#### 3.2.2 Quadratic assignment procedure analysis

QAP is a technique for comparing the similarity of every element in two matrices. It is crucial to explain how “relational” data interacts with and influences one another (35, 36). QAP analysis is based on the permutation of matrix data and may implement the non-parametric test of coefficients (37). This study uses two stages—QAP correlation and QAP regression—to investigate the effects of the emergency collaboration relationships of different modules on the overall JPCM coordination relationship. QAP regression analysis aims to examine the regression relationship between multiple matrices and a single matrix and assess the importance of the decision coefficient R^2^. Thus, a model of the overall JPCM coordination’s influencing mechanism using Equation (8) (38).

(8) $$G=f\left(\mathrm{intr},\mathrm{supl},\mathrm{resc},\mathrm{resp},\mathrm{impl}\right)$$

the independent variables intr,supl,resc,resp,impl stand for the matrices of the emergency collaboration of Interrupt Spread module, Manage Supply module, Medical Rescue module, Restore Work and Production module, and Implement Responsibility module, respectively.

## 4 Case and data

On January 14, 2020, the National Health Commission held a teleconference, proposing for the first time the establishment of a coordinated mechanism for the prevention and control of epidemics, and on January 20, the General Secretary again gave instructions on the comprehensive prevention and control of epidemics. At the executive meeting of the State Council, the Premier proposed a “multi-sectoral joint prevention and control mechanism and emergency plan.” The National Health Commission held a meeting on the same day to convey the spirit of the General Secretary’s instructions and the Premier’s request. The next day, the Joint Prevention and Control Mechanism for the epidemic was formally established. The official documents of the JPCM can be found on its website and the website of the NHSC website. Documents were collected when the website was regularly updated, and new documents can be accessed online. The data was collected from January 2020 to December 2022. Based on the above data, all the information was formatted into a list of edges, generating 1,396 edges. In this study, network diagrams were generated using Gephi 0.0.9 software, network parameters were evaluated using Ucinet 6.232 software, and statistical analysis was performed using SPSS 26.0 software.

## 5 Results

### 5.1 Structure analysis of the JPCM network

**Figure 1:**
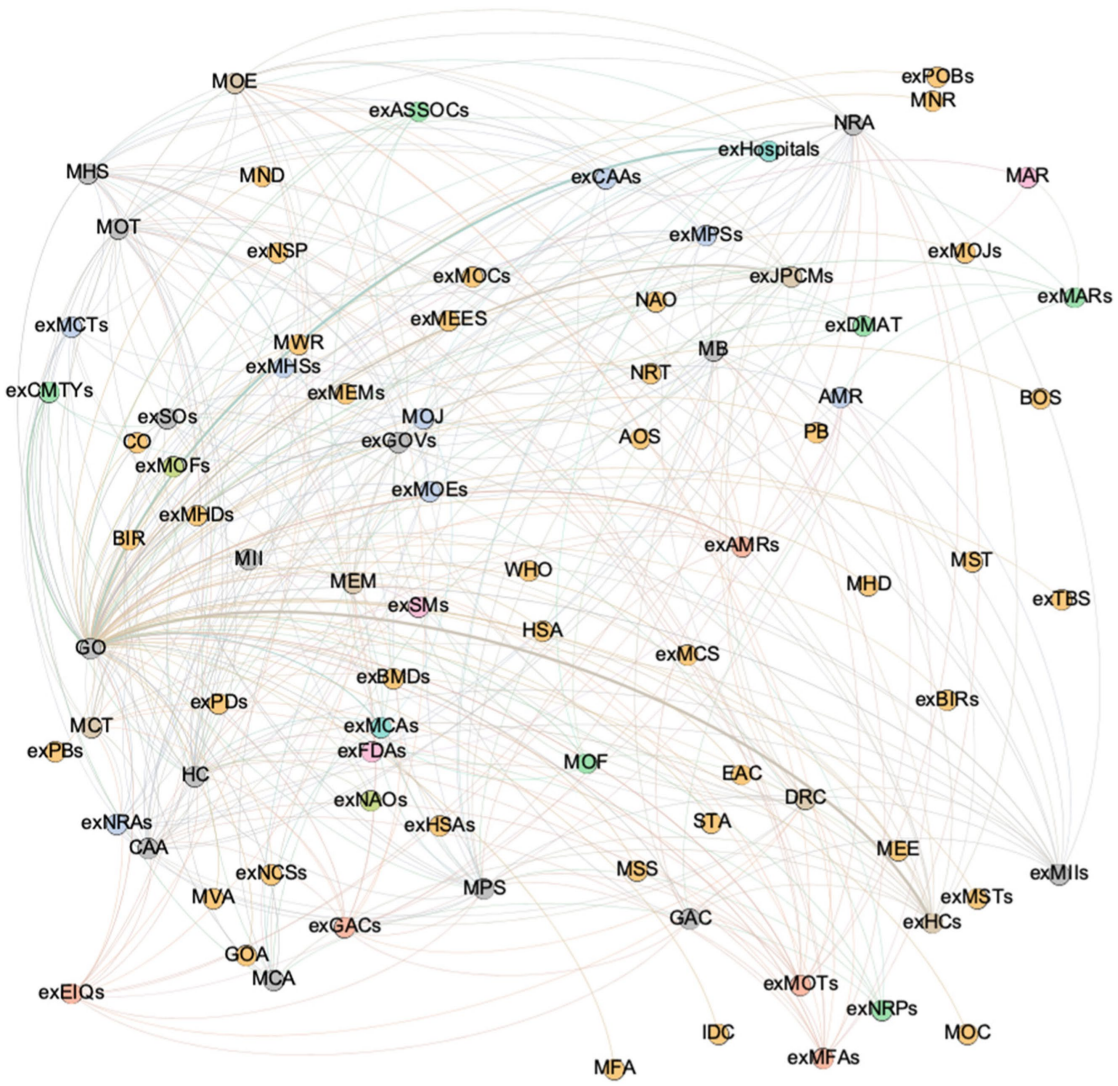
The overall JPCM coordination.

As depicted in Figure 1, the overall network of JPCM was drawn to illustrate the panoramic framework. Where nodes represented the sectors or agencies participating in the network, lines reflected the collaboration between the joint prevention and control group nodes. Meanwhile, the larger the size of the JPCM node, the more nodes were connected to it. In Figure 1, the JPCM of the State Council (JPCM), the JPCMs of provincial and municipal governments (exJPCMs), medical facilities (exHospitals), departments at provincial and municipal levels (exHCs, exMCAs, exMHSs, exMOTs, exMPSs, exMOEs, exCAAs, exNRAs, exMCTs, exMFAs, exMIIs, exAMRs, etc.), the Disaster Medical Assistance Team (exDMAT), and street (township) and community (village) (exCMTYs) were represented by purple, orange, blue, and green nodes.

**Figure 2:**
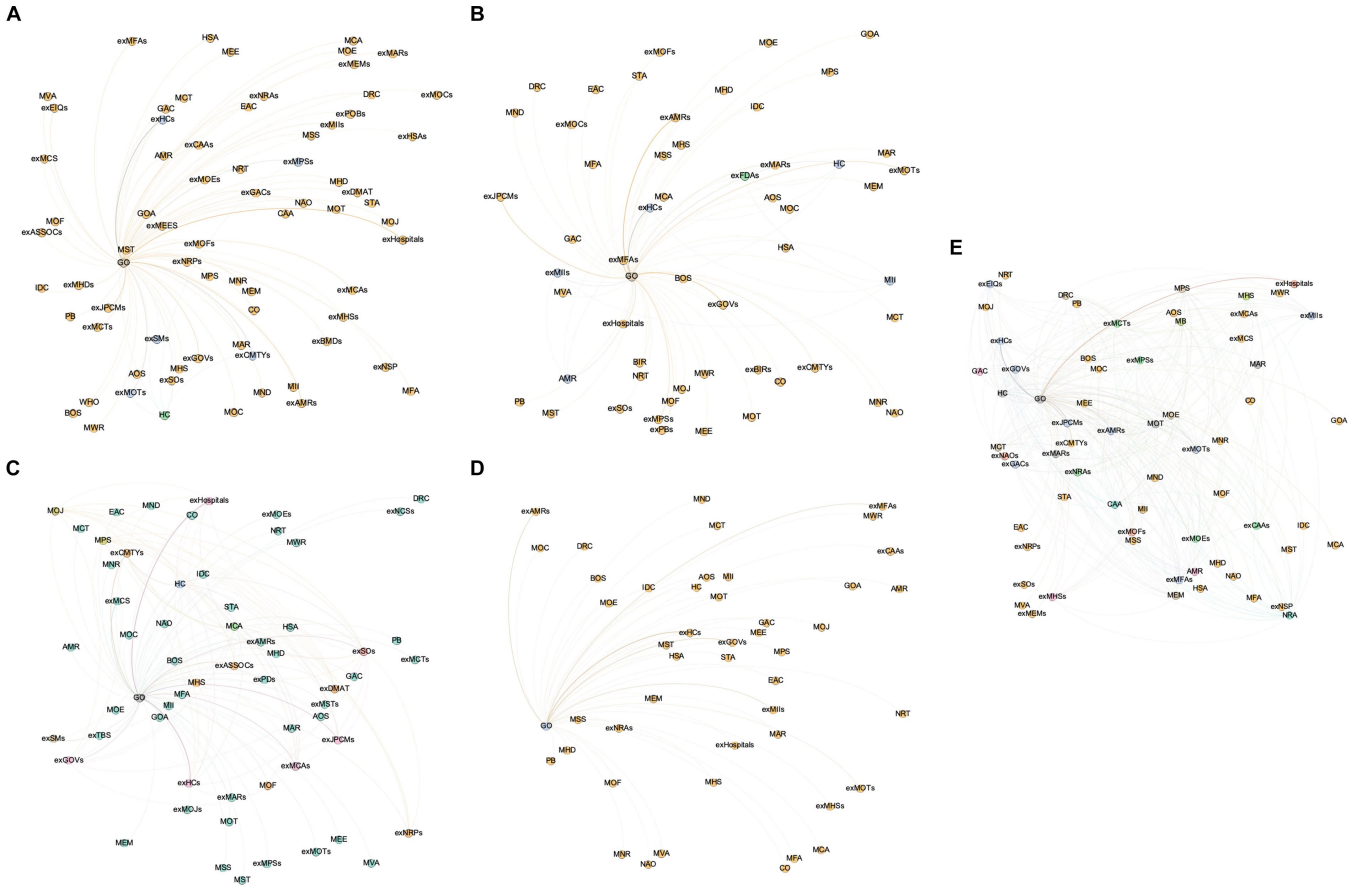
The modularization network of the emergency response. **(A)** The Interrupt Spread module. **(B)** The Manage Supply module. **(C)** The Medical Rescue module. **(D)** The Restore Work and Production module. **(E)** The Implement Responsibility module.

As indicated in Table 1, the network structure indicators were calculated here for each module and the overall JPCM network. The overall network size of the JPCM was 85, indicating that there were a total of 85 departments or agencies participating in the joint control mechanism for the response to the COVID outbreak. Different types of member departments performed different duties in the JPCM and occupy different positions in the collaborative governance network. This study examined the characteristics of different functional working modules. The Interrupt Spread module, Manage Supply module, Medical Rescue module, Restore Work and Production module, and Implement Responsibility module were depicted in Figures 2A–E. As in Figure 1, nodes and lines indicated the participating departments and collaborative relationships. The sectors involved in the Interrupt Spread network are mainly transportation departments, the State Council’s JPCM, and provincial and municipal governments’ exJPCMs. Meanwhile, the exJPCMs of provincial and municipal governments, industry and information technology departments, market regulation departments, and health departments of provincial and municipal governments constituted the fundamental structure of the Manage Supply network. In addition, the health departments, civil affairs departments, and JPCMs of the State Council, as well as these departments at provincial and municipal levels, made up the majority of the departments and agencies in the Medical Rescue network. The representative nodes of the Medical Rescue network also included associations and social organizations for healthcare provision. The representative nodes of the network for the restoration work and production network were the JPCMs and market regulation departments at the State Council and provincial and municipal government levels. Finally, the implementation responsibility network had participating entities such as JPCMs, the State Council’s health departments, emergency management departments, human resources and social insurance departments, and the provincial and municipal governments. The key nodes of the Implement Responsibility network also included market regulation, development and reform, industry and information technology, customs, border inspection, public security, transport, education, and culture and tourism departments of the provincial and municipal governments.

**Table 1:** The structure indicators of the emergency response network.

Indicators	Overall JPCM network	Interrupt spread network	Manage supply network	Medical rescue network	Restore work and production network	Implement responsibility network
# of network size (nodes)	85	73	55	60	47	66
# of collaboration relationships (links)	374	78	62	127	46	286
# of network density (%)	9.7	3.0	4.2	7.1	4.3	13.2
# of network cohesion	0.10	0.03	0.04	0.07	0.04	0.13
# of network centralization (%)	9.51	11.72	23.46	11.04	18.48	11.76
# of average path length	1.94	1.97	1.99	1.93	1.96	1.93
# of network diameter	3	2	3	2	2	3
# of clustering coefficient	0.485	0.762	0.286	0.537	0.000	0.459

The network structure indicators of each module were calculated in Table 1. In the collaborative governance for the COVID pandemic, the Interrupt Spread network, the Manage Supply network, the Medical Rescue network, the Restore Work and Production network, and the Implement Responsibility network had 73, 55, 60, 47, and 66 member departments and agencies, respectively. There were 78, 62, 127, 46, and 286 collaboration partnerships among the member departments and agencies. There was a significant increase in the scale and quantity of the Implement Responsibility module compared to other functional networks. This phenomenon was related to the diversity and complexity of emergency departments and agencies that in the Implement Responsibility functional network. The network densities of the Interrupt Spread, Manage Supply, Medical Rescue, Restore Work and Production, and Implement Responsibility modules were 3.0, 4.2, 7.1, 4.3, and 17.2%, respectively. The Implement Responsibility and Medical Rescue modules had a higher network density, whereas the Interrupt Spread, Manage Supply, and Restore Work and Production modules had relatively lower network densities. This demonstrated that the departments and agencies in the Implement Responsibility and Medical Rescue modules collaborated frequently and had the closest synergistic links. The Interrupt Spread, Manage Supply, and Restore Work and Production modules primarily had collaborative relationships with a relatively low number of departments and agencies; therefore, the overall collaboration density among the departments and agencies was relatively low. Overall, the Implement Responsibility module had a higher network density and network cohesion than the overall JPCM emergency coordination network, indicating a higher degree of sharing among the same emergency response departments and agencies in the Implement Responsibility module network.

The network centralization of the Interrupt Spread, Manage Supply, Medical Rescue, Restore Work and Production, and Implement Responsibility modules was 11.72, 23.46, 11.04, 18.48, and 11.76%, respectively, indicating the presence of prominent core emergency response in the Manage Supply module. Similarly, in the Restore Work and Production network, there was a clear tendency for the network to cluster toward the core emergency response agencies, with a relative concentration of network power. However, in the Interrupt Spread, Medical Rescue, and Implement Responsibility modules, the position of different emergency response departments and agencies was generally balanced, as was the distribution of power among emergency response departments and agencies. The collaboration networks of the Interrupt Spread, Medical Rescue, and Implement Responsibility modules typically exhibited the characteristics of a homogeneous structure.

The network diameters of the Interrupt Spread, Manage Supply, Medical Rescue, Restore Work and Production, and Implement Responsibility modules were correspondingly 2, 3, 2, 2, and 3. Correspondingly, the network average path lengths of the Interrupt Spread, Manage Supply, Medical Rescue, Restore Work and Production, and Implement Responsibility modules were 1.97, 1.99, 1.93, 1.96, and 1.93, which indicated that the Manage Supply module had the best collaboration network connectivity, the collaboration and communication distance between the departments and agencies was the shortest, and collaboration between the departments and agencies was more consistent. Despite having a network diameter of 3, the Implement Responsibility module’s average path length was also low. This indicated that the implemented responsibility collaboration network had convenient connectivity and that communication between the emergency agencies was efficient and smooth.

The clustering coefficients for the modules of Interrupt Spread, Manage Supply, Medical Rescue, Restore Work and Production, and Implement Responsibility were 0.762, 0.286, 0.537, 0.000, and 0.459, respectively. Therefore, the emergency departments and agencies involved in the Interrupt Spread and Medical Rescue modules had established a number of stable and cooperative links. The emergency departments and agencies with a cooperating connection had higher agglomeration levels. Additionally, the clustering coefficients of the Interrupt Spread and Medical Rescue modules were higher than those of the overall JPCM network, illustrating that the JPCM network had not yet established a stable clustering organization model. However, due to the high degree of heterogeneity among the emergency departments and agencies in the network, all of them were more likely to have access to heterogeneous emergency resources and had higher emergency resource transfer efficiency.

### 5.2 Cross-regional characteristics of networks

The E–I index was introduced to explore the resource flow and faction structure among departments and agencies in the emergency response network. To assess the E-I index’s effectiveness and determine whether each network’s E-I index is random, we performed 5,000 random permutations of the same nodes and relational links. According to Table 2, the overall JPCM and Implement Responsibility networks passed with a value of *p* < 0.001, while the Manage Supply and Medical Rescue networks passed the significance test with *p* < 0.05. Therefore, the E-I index observations for the four networks were statistically significant.

**Table 2:** Observed E–I index with random permutations.

`	Observed E–Iindex	Min E–I index in permutations	Average E–I index in permutations	Max E–I index in permutations
Overall JPCM network	−0.192^***^	−0.231	0.01	0.276
Interrupt spread network	0.013	−0.091	0.012	0.117
Manage supply network	−0.452^*^	−0.484	−0.132	0.516
Medical rescue network	0.122^*^	−0.545	−0.038	0.382
Restore work and production network	−0.565	−0.565	−0.326	0.609
Implement responsibility network	0.147^***^	−0.477	−0.03	0.29

^*^p < 0.05, ^**^p < 0.01, and ^***^p < 0.001.

According to Table 2, the E–I index for the Medical Rescue and Implement Responsibility networks were 0.122 and 0.147, respectively. The finding suggested that there were more external relationships than internal ones. Cross-group emergency response coordination was at a higher level. In addition, it was found that within the Medical Rescue and Implement Responsibility networks, different departments and agencies had a stronger sense of identity and were better able to respond to emergencies. In contrast, the E–I indexes for the overall JPCM and Manage Supply networks were − 0.192 and − 0.452, respectively. This showed that each group of departments and agencies in these networks was likelier to engage in intra-group than inter-group collaboration.

**Figure 3:**
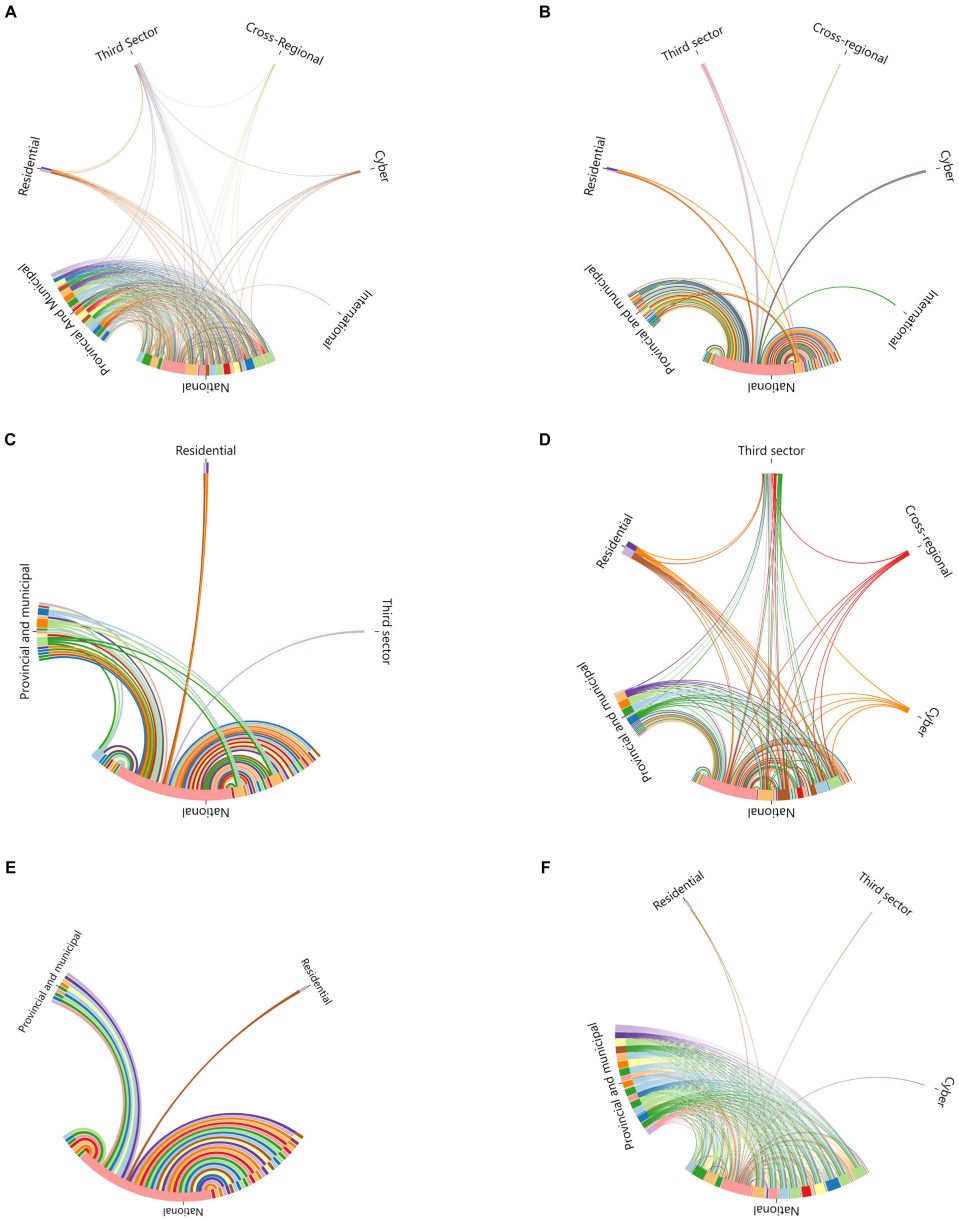
Chord diagrams of the relationships between groups. **(A)** The overall JPCM coordination. **(B)** The Interrupt Spread module. **(C)** The Manage Supply module. **(D)** The Medical Rescue module. **(E)** The Restore Work and Production module. **(F)** The Implement Responsibility module.

Further, the external and internal links were calculated for each group of departments and agencies. Drawing chord diagrams of the relationships between groups of departments and agencies (see Figures 3A–F) allowed us to discuss the collaborative relationships, frequency of interactions, and resource transfers between groups of departments and agencies in the overall JPCM network and in the different emergency response modules. The E-I indices for each group of departments and agencies in the overall JPCM network and the Interrupt Spread, Manage Supply, Medical Rescue, Restore Work and Production, and Implement Responsibility modules were given in Tables 3–6 (networks with statistically significant E-I indices).

**Table 3:** Interactions in the overall JPCM network.

Group	Internal links	External links	Total links	E–I index
International	0	1	1	1.000
National	245	173	418	−0.172
Provincial and municipal	149	100	249	−0.197
Cross-regional	4	2	6	−0.333
Residential	10	4	14	−0.429
Third sector	17	6	23	−0.478
Cyber	3	4	7	0.143

**Table 4:** Interactions in the manage supply module.

Group	Internal links	External links	Total links	E–I index
International	0	0	0	/
National	90	34	124	−0.452
Provincial and municipal	12	10	22	−0.091
Cross-regional	0	0	0	/
Residential	0	2	2	1.000
Third sector	0	1	1	1.000
Cyber	0	0	0	/

**Table 5:** Interactions in the medical rescue module.

Group	Internal links	External links	Total links	E–I index
International	0	0	0	/
National	57	96	153	0.255
Provincial and municipal	26	18	44	−0.182
Cross-regional	4	2	6	−0.333
Residential	6	8	14	0.143
Third sector	13	10	23	−0.130
Cyber	2	4	6	0.333

**Table 6:** Interactions in the implement responsibility module.

Group	Internal links	External links	Total links	E–I index
International	0	0	0	/
National	133	209	342	0.222
Provincial and municipal	103	107	210	0.019
Cross-regional	0	0	0	/
Residential	2	1	3	−0.333
Third sector	0	1	1	1.000
Cyber	0	2	2	1.000

Table 3 displayed the external and internal links and E–I index for several groupings within the overall JPCM network. The E-I index for the international group was 1. International cooperation within the JPCM network referred to the link with the World Health Organization (WHO), which notified the epidemic and, in January 2020, established a support team to respond to the early outbreak. Therefore, the international group’s partnership with the JPCM network was an external link. The national, provincial and municipal E-I indexes are −0.172 and −0.197, respectively. This suggested that although the national group issued orders to the provincial and municipal groups, and the provincial and municipal groups were then required to receive and execute those orders, there were slightly more collaborative relationships within the same jurisdiction than between the external emergency response agencies of these groups. The E-I indices for the cross-regional, residential, and third-sector groups were −0.333, −0.429, and −0.478, respectively. This indicated that the departments and agencies within these groupings were more inclined to collaborate internally and provide resources. In the JPCM network, the cross-regional groups mainly referred to the Disaster Medical Assistance Team (Emergency Medical Teams (EMTs) as classified by WHO), which was dispatched to the center of a pandemic to assist in health emergencies. Residential groups consisted of local hospitals, communities, and neighborhoods. After receiving direction and resources from external relationships, the residential group primarily engaged in emergency response coordination through internal operations. Moreover, this also occurred in the third sector group. In addition, the cyber group mainly coordinated with external offline emergency response organizations.

Table 4 demonstrated the external/internal links and E–I index for each group in the Manage Supply module. The E-I index of the national group was −0.452, indicating that national emergency response organizations were more inclined to engage in internal collaboration and resource provision in the Manage Supply module. The E-I index of provincial and municipal organizations was −0.091, indicating that their synergistic relationship with external emergency organizations was almost random. In the Manage Supply module, the E-I index for the residential and the third sector group was 1, suggesting that residential and online departments and agencies relied exclusively on external collaboration and resource provision.

Table 5 presented the external/internal links and E–I index for each group in the Medical Rescue network. The E–I index of a national group was 0.255, which can improve the coordination and direction of heterogeneous nodes in the Medical Rescue collaborative network. The E–I index of the provincial and municipal group was −0.182, indicating that provincial and municipal emergency agencies preferred internal collaboration and resource provision for Medical Rescue tasks. Furthermore, the E-I indexes of the cross-regional and third sector were − 0.333 and − 0.130, respectively, indicating that when it came to Medical Rescue tasks, information and resources flew more within the cross-regional and third sector groups. In addition, the E–I indexes for the residential and cyber groups were 0.143 and 0.333, respectively. This indicated that the residential and cyber agencies were connected to external nodes for obtaining resources and carrying out rescue activities.

The external/internal links and E–I indices of the groups in the implement responsibilities network were shown in Table 6. The E-I index of the national group was 0.222, and that of the provincial group was 0.019. This was because the activities of receiving administrative instructions and creating a connection between the diverse nodes in the implement responsibilities module were primarily moved from higher-level to lower-level departments. The E-I index for the residential group was −0.333, indicating that more internal nodes were responsible for improving the implementation of emergency management at the residential level. The E-I index for both the third sector and cyber groups was 1. The collaborative relationships between the third sector and cyber participants in the implement responsibilities module were provided exclusively by external entities of administrative power.

### 5.3 The influence of the modular networks on the overall JPCM network

The JPCM emergency response coordination model was diverse and had complex tasks, including interrupting the spread of the epidemic, managing the logistics and supply of emergency supplies, providing medical care for infected people, safeguarding economic stability and the order of work and production, and reinforcing the implementation of instructions in all segments. The emergency coordination model consisted of several emergency function modules, which affected the construction and development of JPCM’s overall emergency coordination network. For this reason, QAP analysis was applied to study each emergency response functional module in JPCM emergency response coordination to elucidate each module’s driving mechanism and influence on the construction of overall emergency response coordination.

#### 5.3.1 QAP correlation analysis

In this study, 5,000 permutations were used to investigate the link between the overall JPCM emergency response coordination matrix and each module’s emergency response coordination matrix. The correlation coefficients between the overall JPCM collaboration matrix and each module’s collaboration matrix were displayed in Table 7. The correlation analysis results showed a significant correlation between the collaboration matrix of each emergency response module and the overall JPCM matrix, with a significance level of *p* < 0.001. The correlation coefficients corresponding to the Interrupt Spread, Manage Supply, Medical Rescue, Restore Work and Production, and Implement Responsibility modules were 0.985, 0.636, 0.898, 0.559, and 0.956, respectively. It can be seen that the Interrupt Spread, Manage Supply, Medical Rescue, Restore Work and Production, and Implement Responsibility modules all positively influence the development of the overall JPCM emergency coordination network.

**Table 7:** QAP correlation analysis results.

Functional network	Observed value	Std. dev.
Interrupt spread	0.985^***^	0.029
Manage supply	0.636^***^	0.036
Medical rescue	0.898^***^	0.026
Restore work and production	0.559^***^	0.034
Implement responsibility	0.956^***^	0.031

^*^p < 0.05, ^**^p < 0.01, and ^***^p < 0.001.

#### 5.3.2 QAP regression analysis

A QAP regression analysis using 2,000 random displacements was performed to test the effects of each module’s matrix on the total JPCM matrix. The results were given in Table 8. The regression model based on the Interrupt Spread, Manage Supply, Medical Rescue, Restore Work and Production, and Implement Responsibility matrix strongly explained the overall JPCM matrix, with a value of 0.993 (r-squared) for the QAP regression test. This demonstrated that a change in the Interrupt Spread, Manage Supply, Medical Rescue, Restore Work and Production, and Implement Responsibility modules would change the overall JPCM network. In addition, the matrix of the Interrupt Spread, Medical Rescue, and Implement Responsibility modules had a positive effect on the formation of the overall JPCM matrix, i.e., an increase in the variance of the collaborative network of any of the three individual emergency response modules would increase the variance of the overall JPCM network. There was a negative effect of the emergency response coordination matrices of the Manage Supply and the Restore Work and Production modules, but all passed the significance test at *p* < 0.01.

**Table 8:** Regression analysis results.

Variable	Unstd.-ized coefficient	Std.-ized coefficient	
Intercept	−0.018989	0.000	
Interrupt spread	1.408^***^	0.664	
Manage supply	−0.532^***^	−0.035	
Medical rescue	0.778^***^	0.198	
Restore work and production	−0.557^***^	−0.035	
Implement responsibility	0.803^***^	0.212	
R-square	0.993	Adj R-Sqr	0.993

^*^p < 0.05, ^**^p < 0.01, and ^***^p < 0.001.

In this case, the most vital driver on the overall JPCM network was the Interrupt Spread module, with a standardized regression coefficient of 0.664. The following two modules were the Implement Responsibility and Medical Rescue modules, with standardized regression coefficients of 0.212 and 0.198, respectively. These results suggested that the emergency response coordination generated by the Interrupt Spread, Implement Responsibility, and Medical Rescue modules was the major factor in the increased scope and variability of the JPCM emergency coordination. However, the matrixes of the Manage Supply and Restore Work and Production modules were negatively associated with the development of JPCM collaboration.

## 6 Discussion

In recent years, due to the complexity of health emergencies and the establishment of emergency management operational systems, emergency management practice has taken on a distinctly cross-organizational character. From this point of view, the study in this paper extended the theme of cross-organizational collaboration in previous emergency management literature (14–16, 19). From a network-centric vantage point, this study explored the synergy between the emergency collaboration networks of the various functional modules and the overall JPCM network in the case of China’s COVID outbreak prevention and control. When responding to COVID, departments and agencies from different jurisdictions have worked together. However, they had a unique collaborative approach and coordinated their limited resources. Additionally, the construction of the overall JPCM emergency response coordination network was influenced by the role of the synergistic network of each emergency functional module interaction.

The JPCM collaboration network began as a cross-organizational collaborative practice through the Interrupt Spread module and established a holistic emergency response coordination with most emergency response departments and agencies (39). We found that much of the literature on China’s epidemic prevention and control focused on transportation and logistics measures to interrupt the spread of epidemics (6, 11) This manuscript emphasized the role of the Interrupt Spread module. The synergistic relationship of the Interrupt Spread module had the most positive impact on establishing the JPCM emergency response coordination network. The Interrupt Spread module enabled the activation of the overall efficiency of JPCM collaboration.

The key to the Manage Supply module was to ensure the flow of medical supplies and survival necessities (40, 41). To this end, the departments and agencies involved in the collaboration in the Manage Supply module were closely linked. It was highly centralized, although in this case, the involvement of the Manage Supply collaboration network was smaller, for example, the Manage Supply network was centrally dominated by the emergency response departments of the national, provincial, and municipal emergency response departments. This study stood out from previous emergency management studies by using a diagrammatic approach to illustrate the specific situations in which departments collaborate in emergency supply security and how this collaboration developed (42). It was also discovered through this study that based on managing the delivery of supply collaboration, emergency organizations might be more cohesive in their coordination areas within the overall JPCM network, and the overall collaborative relationship could be strengthened.

The Medical Rescue module established a diversified and intensive collaboration model between governmental, third sector, residential, and cyber emergency organizations (43). This was because the Medical Rescue process addressed many core aspects of a health emergency response, such as early disposal, infected treatment, vaccine management, and on-site vigilance, which continued throughout the pandemic response (44). This study found that the E–I indexes of the national, residential, and cyber groups in the Medical Rescue collaboration network were > 0, and the E–I indexes of the provincial and municipal, cross-regional, and third sector groups in the Medical Rescue collaboration network were < 0. This meant that in the Medical Rescue functional module, departments and agencies of the national, community, and cyber groups could not realize their functions through intra-group links. They had to send out-of-group links to realize in-group cooperation in the provincial, municipal, cross-regional, and third sectors. From this perspective, the Medical Rescue module broadened the JPCM network framework regarding emergency organizational relationships and collaboration densities. Therefore, it significantly impacted the creation of JPCM collaborative relationships.

Restore Work and Production includes functional emergency organizations centered on restoring production, stabilizing employment and the economy, and sustaining routine work after the epidemic (45, 46). The departments and agencies involved in the collaboration were centralized, and the delivery of the Restore Work and Production module allowed for the increase and development of the scope and depth of synergies between emergency response organizations on modules of the Interrupt Spread, Medical Rescue, Manage Supply, and Implement Responsibility modules could be increased and developed.

In the Implement Responsibility module, the different groups of functional emergency departments and agencies have established a rich synergy around the assurance aspects of the Interrupt Spread, Medical Rescue, Manage Supply, and Restore Work and Production functional networks, which comprised essential support for the effective transmission and stable operation of the overall JPCM network and the achievement of the ultimate efficiency of the overall emergency response coordination (47). JPCM’s GO leadership organization often issued red-tape documents titled “Responsibility for Implementation” (39–41, 43, 46). In this paper, through network analysis, we further concluded that the network density, network concentration and network cohesion of the Implement Responsibility coordination network were larger; in the chord diagram analysis, we also noted that the Implement Responsibility module provided more heterogeneous emergency response resources for the overall JPCM coordination network, which was an important complement to the COVID cross-organizational emergency response coordination. Finally, the regression analysis in this text found that the Implement Responsibility module positively impacted the formation of the JPCM collaborative network.

## 7 Policy recommendations

Initially, when responding to sudden public crisis events, it is imperative to establish multiple working groups with distinct professional duties. For example, in the context of COVID in China, in addition to the departments that sustained the medical rescue, the joint efforts of working groups on, for example, transportation, materials, and economic life are needed. Simultaneously, due to their extensive scope, these working groups can be shared by some functional departments to carry out specific tasks related to incident response. For instance, during the COVID outbreak in China, multiple functional working groups incorporated the efforts of the transportation, health and finance departments.

Furthermore, it is imperative to establish a central command structure that would facilitate communication with various working groups, give consistent directives, and coordinate the efforts of many working groups. Due to the various institutional and cultural settings, this effort can also be reflected in official documents/decrees that enhance emergency response implementation. In the case of COVID in China, the GO of JPCM assumed the role (the leading group) Additionally, the issuance of the JPCM’s Implementation Responsibility document also played a crucial role in enhancing the coordination of emergency response efforts across various regions and strengthening the overall collaborative relationship.

Lastly, medical rescue efforts require the establishment of cooperative frameworks involving the government, the non-profit sector, and the local community. The COVID situation in China demonstrates the need to strengthen multi-party dialogue among the medical industry, government, and community to maximize the efficiency and efficacy of medical rescue activities.

## 8 Conclusion and limitations

This study used social network techniques to identify collaborative behaviors among emergency response organizations in China’s Joint Prevention and Control Mechanism (JPCM) working groups. The COVID health emergency response capacity was assessed under the analytical framework of modularization of emergency collaboration network and integration of overall emergency response network. The findings of the study can provide guidance for improving public health emergency management and developing cross-organizational public health emergency management. In addition, the study can assist emergency management organizations in making sensible emergency judgments in light of various emergency response functions. It can also be used to help emergency management organizations in making informed decisions in various emergencies.

This study had limitations. On the one hand, it focused on macro-level mechanisms, although the involvement of local groups of emergency organizations in prevention and management was noted. There is a need to integrate more examples in the following research. On the other hand, this study explored emergency coordination from the perspective of functional emergency response networks. Nonetheless, there is a need to investigate the structural characteristics further and influencing factors of cross-organizational collaborative networks from a temporal perspective, taking into account the full life cycle characteristics of emergency response networks.

## Data availability statement

Publicly available datasets were analyzed in this study. This data can be found at: https://www.gov.cn/zhengce/gwylflkjzwj.htm.

## Ethics statement

This research does not require ethical approval because it utilizes accessible public information. And informed consent for further analysis of datasets has been obtained from disclosure when the original data was collected.

## Author contributions

The author confirms being the sole contributor of this work and has approved it for publication.
