# Supplementary material for: Coordination and modularization: the experience of the joint prevention and control mechanism to COVID emergencies in China
Source: Front Public Health. 2024 Apr 11;12:1244769. doi: 10.3389/fpubh.2024.1244769 (PMC11043556; doi:10.3389/fpubh.2024.1244769)
Supplement: Supplementary file 1 [file Table_1.docx]

Appendix

Table A1 Departments involved in JPCM emergency response.

| Rank | Name | Abbreviation |
| --- | --- | --- |
| 1 | Ministry of Foreign Affairs | MFA |
| 2 | Development and Reform Commission | DRC |
| 3 | Ministry of Science and Technology | MST |
| 4 | Ethnic Affairs Commission | EAC |
| 5 | Ministry of State Security | MSS |
| 6 | Ministry of Justice | MOJ |
| 7 | Ministry of Human Resources and Social Security | MHS |
| 8 | Ministry of Ecology and Environment | MEE |
| 9 | Ministry of Transport | MOT |
| 10 | Ministry of Agriculture and Rural Affairs | MAR |
| 11 | Ministry of Culture and Tourism | MCT |
| 12 | Ministry of Veterans Affairs | MVA |
| 13 | People's Bank | PB |
| 14 | Ministry of National Defense | MND |
| 15 | Ministry of Education | MOE |
| 16 | Ministry of Industry and Information Technology | MII |
| 17 | Ministry of Public Security | MPS |
| 18 | Ministry of Civil Affairs | MCA |
| 19 | Ministry of Finance | MOF |
| 20 | Ministry of Natural Resources | MNR |
| 21 | Ministry of Housing and Urban-Rural Development | MHD |
| 22 | Ministry of Water Resources | MWR |
| 23 | Ministry of Commerce | MOC |
| 24 | Health Commission | HC |
| 25 | Ministry of Emergency Management | MEM |
| 26 | National Audit Office | NAO |
| 27 | General Administration of Customs | GAC |
| 28 | State Administration for Market Regulation | AMR |
| 29 | General Administration of Sports | AOS |
| 30 | International Development Cooperation Agency | IDC |
| 31 | Counsellor's Office | CO |
| 32 | State Taxation Administration | STA |
| 33 | National Radio and Television | NRT |
| 34 | Bureau of Statistics | BOS |
| 35 | National Healthcare Security Administration | HSA |
| 36 | National Governmental Offices Administration | GOA |
| 37 | Meteorological Bureau | MB |
| 38 | Banking and Insurance Regulatory Commission | BIR |
| 39 | National Railway Administration | NRA |
| 40 | Civil Aviation Administration | CAA |
